# Supplementary material for: The role of shear in crystallization kinetics: From suppression to enhancement
Source: Sci Rep. 2015 Sep 29;5:14610. doi: 10.1038/srep14610 (PMC4586493; doi:10.1038/srep14610)
Supplement: Supplementary Tables and Figures [file srep14610-s1.pdf]

Supplementary figures

The role of shear in crystallization kinetics: From suppression to enhancement

David Richard and Thomas Speck

Institut für Physik, Johannes Gutenberg-Universität Mainz, Staudingerweg 7-9, 55128 Mainz, Germany

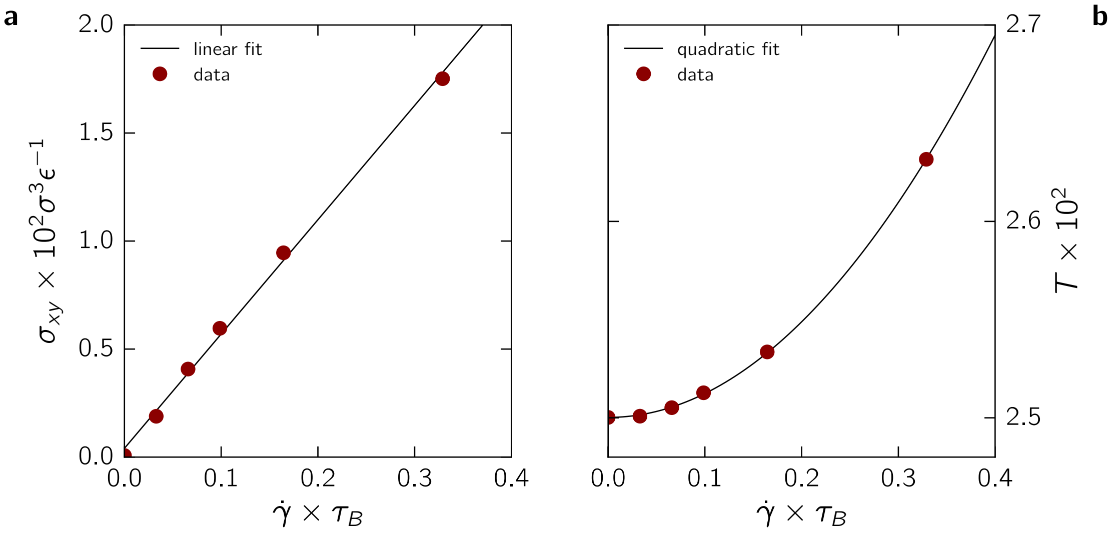

Supplementary Figure 1. *Linear response regime.* Shown is the response of the metastable liquid in the middle of the coexistence region ($\phi \simeq 0.525$). (a) Off-diagonal component

$$\sigma_{xy} = -\frac{1}{V}\langle \sum_{i=1}^{N}[m_i v_{i,x} v_{i,y} + \sum_{j\neq i} r_{ij,x} F_{ij,y}]\rangle$$

of the stress tensor as a function of the strain rate $\dot{\gamma}$. It shows a linear behavior and corresponds to the Newtonian regime, where $\sigma = \eta\dot{\gamma}$ with η the shear viscosity. (b) Kinetic temperature against the strain rate. In our simulations we employ mostly strain rates $\dot{\gamma}\tau_{\mathrm{B}} < 0.1$. For these low strain rates the temperature of the metastable liquid remains equal to the one of the thermal bath ($T = 0.025$). The solid lines are linear and quadratic fits, respectively.

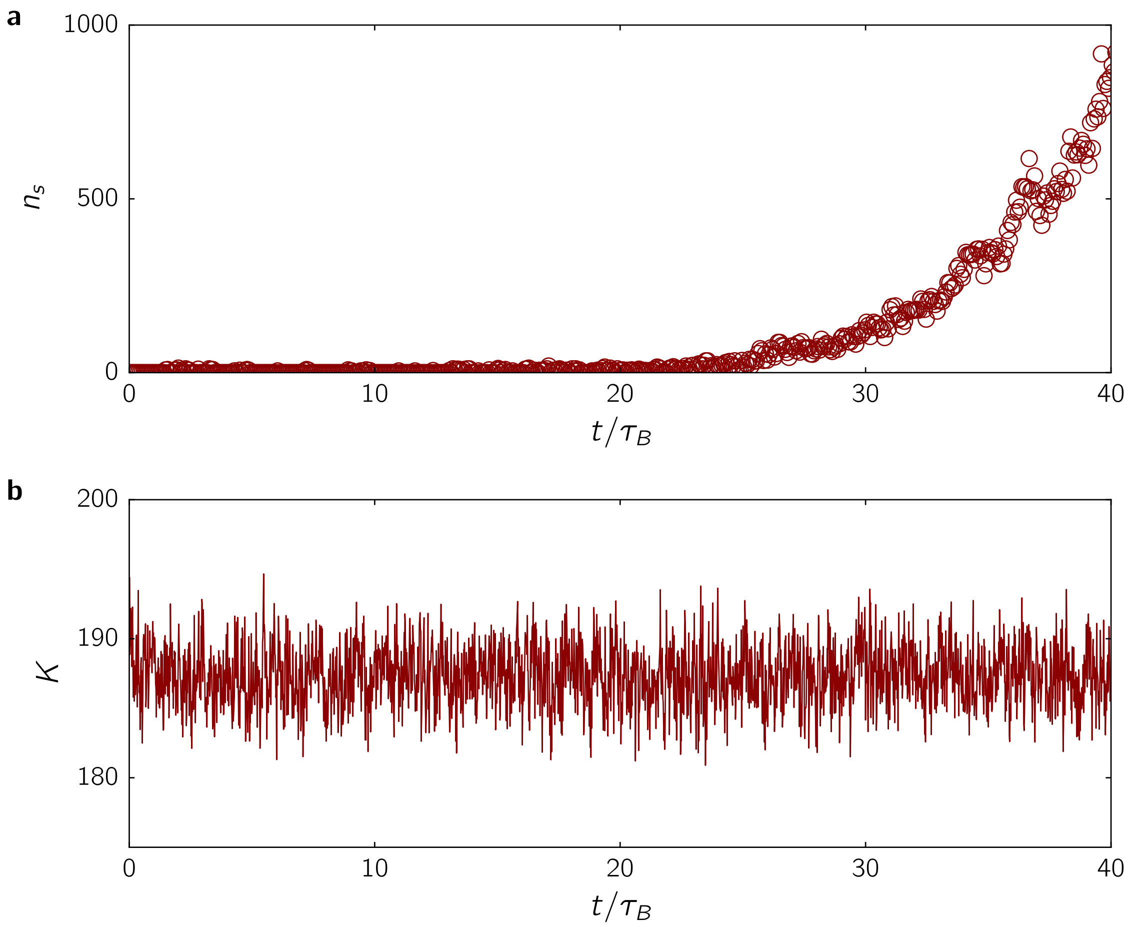

Supplementary Figure 2. *Fluctuations of the kinetic energy during crystallization.* (a) Number of particles in the largest solid cluster during the crystallization growth for $\phi \simeq 0.542$ as a function of time for a single run. (b) The kinetic energy of the system during the growth shows no sign of the crystallization, which indicates that latent heat plays no role for the kinetics.

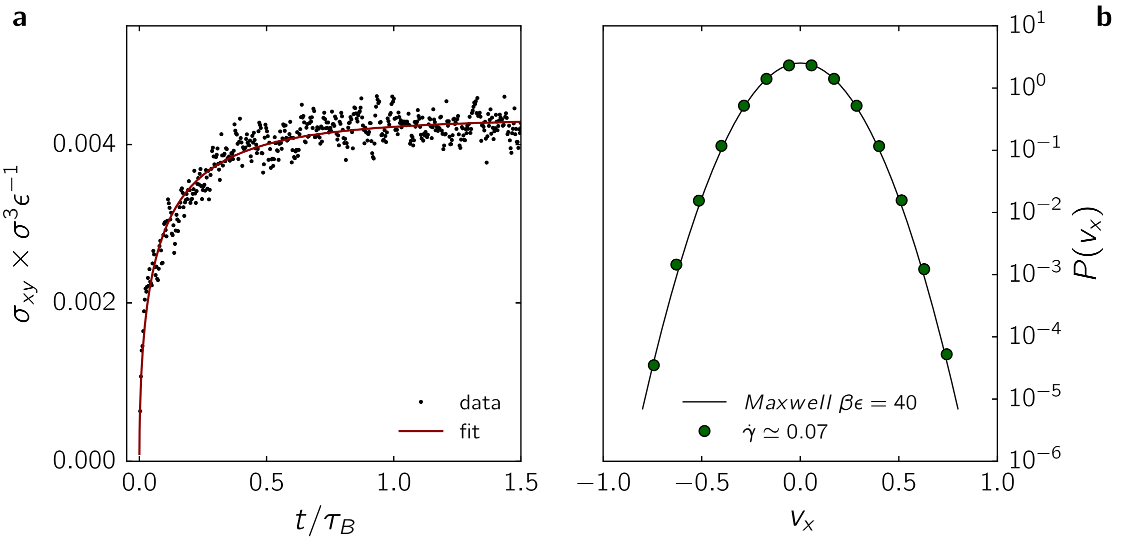

Supplementary Figure 3. *Response to shear.* (a) Shown is the temporal evolution of the stress σ_{xy} after suddenly switching on shear flow with strain rate $\dot{\gamma} \simeq 0.07$ (the same strain rate used in the committor analysis). The dots are averaged over 500 independents runs. The solid line corresponds to a stretched exponential fit of the form

$$\sigma_{xy}(t) = \sigma_{xy}^{ss}(1 - e^{-(t/\tau)^{\beta}})$$

with steady shear stress σ_{xy}^{ss}, relaxation time $\tau \simeq 0.08\tau_{\rm B}$, and stretching exponent $\beta = 0.52$. Hence, we conclude that for the committor analysis the response time τ is negligible when using a Maxwell-Boltzmann distribution for the velocities compared to the fleeting time $\tau_f \simeq 18\tau_B$. (b) Comparison of the velocity distribution for the sheared fluid (symbols) with the equilibrium Maxwell-Boltzmann distribution for $\beta\epsilon = 40$ (line). For low strain rates $\dot{\gamma} < 0.1$ the deviation from the equilibrium Gaussian distribution is negligible.
